# Mesenchymal Stem Cells in Primary Sjögren's Syndrome: Prospective and Challenges

**DOI:** 10.1155/2018/4357865

**Published:** 2018-09-16

**Authors:** Weiqian Chen, Ye Yu, Jilin Ma, Nancy Olsen, Jin Lin

**Affiliations:** ^1^Division of Rheumatology, the First Affiliated Hospital, College of Medicine, Zhejiang University, Hangzhou, 310003 Zhejiang Province, China; ^2^Division of Nephrology, Zhejiang Traditional Chinese Medicine and Western Medicine Hospital, Hangzhou, 310000 Zhejiang Province, China; ^3^Division of Rheumatology, Department of Medicine, Penn State University Hershey College of Medicine Hershey, 17033, USA

## Abstract

Primary Sjögren's syndrome (pSS) is a chronic systemic inflammatory autoimmune disease characterized by lymphocytic infiltrates in exocrine glands. Current approaches do not control harmful autoimmune attacks or prevent irreversible damage and have considerable side effects. Mesenchymal stem cells (MSCs) have been effective in the treatment of several autoimmune diseases. The objective of this review is to illustrate the potential therapeutic role of MSCs in pSS. We summarize the recent advances in what is known about their immunomodulatory function and therapeutic applications in pSS. MSC transfusion can suppress autoimmunity and restore salivary gland secretory function in mouse models and patients with pSS by inducing regulatory T cells, suppressing Th1, Th17, and T follicular helper cell responses. In addition, MSCs can differentiate into salivary epithelial cells, presenting an option as a suitable alternative treatment. We also discuss current bioengineering methods which improve functions of MSCs for pSS. However, there remain many challenges to overcome before their wide clinical application.

## 1. Introduction

Primary Sjögren's syndrome (pSS) is a chronic, systemic autoimmune disease characterized by lymphocytic infiltrates in salivary and lacrimal glands which lead to the destruction of these glands. It affects globally 0.05–1% of people, with manifestations including xerostomia (dry mouth), dental caries, and xerophthalmia (dry eye) [1]. Activated B lymphocytes are another hallmark of the disease [2]; many antibodies appear in the circulation and tissues. Accordingly, systemic extraglandular involvement is common, including synovitis, interstitial lung disease, neuropathy, renal disease, vasculitis, and autoimmune cytopenias [3]. Furthermore, approximately 5–10% of patients may develop lymphoma, mainly the mucosa-associated lymphoid tissue non-Hodgkin lymphoma, which represents the most severe complication of the disease [4]. Although the exact etiology is unclear, it is known that adaptive and innate immune cell imbalances are involved in the pathogenesis of pSS [5–7]. Current approaches such as traditional disease-modifying antirheumatic drugs and biologic agents do not cure this disease and have considerable side and toxic effects [8]. Thus, the development of novel treatments is critically important for pSS.

Mesenchymal stem cells (MSCs), a group of mesodermal and ectodermal origin multipotent stromal cells, are first discovered by Friedenstein et al. [9]. MSCs have a capacity of self-renewal and differentiation into osteoblasts, adipocytes, and chondrocytes [10, 11]. They are of interest due to their rapid proliferation *in vitro* and strong immunomodulation [12]. Notably, MSCs have been successfully isolated from almost all adult tissues, including bone marrow, umbilical cord blood, adipose tissue, dental tissue, skin, and placenta [13–17]. Until now, bone marrow MSCs (BMSCs) and umbilical cord MSCs (UMSCs) have been most widely studied. Subsequently, other types of MSCs are reported, such as gingiva-derived MSCs (GMSCs) and adipose-derived MSCs (AMSCs). Unlike MSCs in bone marrow and umbilical cord blood, GMSCs and AMSCs are both abundant and easily accessible, and they can often be obtained as a discarded biological sample following dental procedures or abdominal surgery. GMSCs and AMSCs are relatively easy to isolate, homogenous and proliferate rapidly *in vitro* [18]. Interestingly, no tumor is observed in the mice which are injected with GMSCs. It indicated GMSCs are nontumorigenic [19]. AMSCs also show a low tendency to develop a tumor [20].

Here, we describe the therapeutic role of MSCs in pSS based on recent relevant publications. Indeed, MSCs have been effective in treating autoimmune diseases such as systemic lupus erythematosus, rheumatoid arthritis, systemic sclerosis, and type 1 diabetes mellitus. Moreover, these treatments have no significant side effects [21–27]. Several years ago, scientists summarized the preliminary studies of MSC treatment for salivary gland dysfunction and xerostomia [28, 29]. A recently published review focuses on MSCs for treating autoimmune dacryoadenitis but not the other aspects of pSS [30]. Existing evidence supports the crucial role of MSCs in the treatment of animal models and patients with pSS. MSCs may also differentiate into salivary epithelial cells, presenting an option as a suitable alternative treatment [31, 32]. In this review, we summarize the immunomodulatory effects of MSCs both in the adaptive and the innate immune responses. The defective function of MSCs in pSS is then discussed, followed by a summary of the use of MSCs in the treatment of patients with pSS or animal models. Finally, the role of bioengineering in enhancing MSC treatment is discussed.

## 2. Immunomodulatory Properties of MSCs on Adaptive and Innate Immune Responses

**Figure 1:**
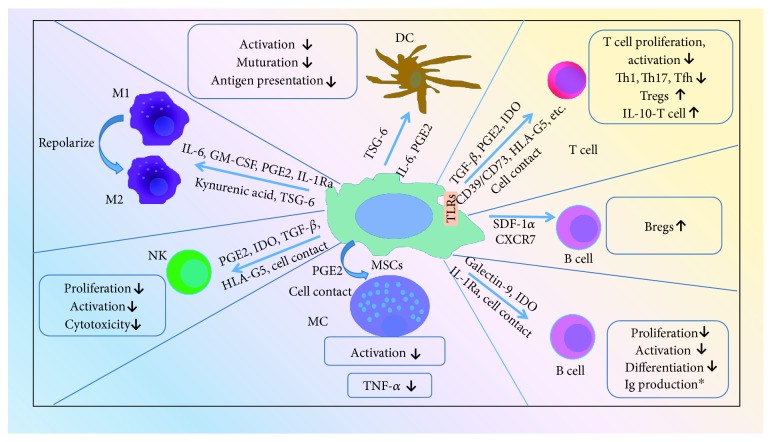
Immunomodulatory properties of mesenchymal stem cells. DC: dendritic cells; TSG-6: TNF-*α*-stimulated gene 6; PGE2: prostaglandin E2; M1: classically activated macrophages; M2: alternatively activated macrophages; GM-CSF: granulocyte-macrophage colony stimulating factor; IL-1Ra: IL-1 receptor antagonist; NK: natural killer T cells; IDO: indoleamine 2,3-dioxygenase; TGF-*β*: transforming growth factor-beta; HLA-G5: human leukocyte antigen-G5; MC: mast cells; MSCs: mesenchymal stem cells; Th: T helper; Tfh: T follicular helper cell; Tregs: regulatory T cells; TLRs: Toll-like receptors; SDF-1*α*: stromal derived factor-1*α*; CXCR7: CXC chemokine receptor 7; Bregs: regulatory B cells; ^∗^: controversial.

The most attractive property of MSCs is their immunosuppression on both adaptive and innate immune responses. MSCs exert major immunomodulatory effects through cell to cell contact and release of soluble factors such as prostaglandin E2 (PGE2), indoleamine 2,3-dioxygenase (IDO), nitric oxide, transforming growth factor-beta (TGF-*β*), human leukocyte antigen-G5 (HLA-G5), CD39/CD73, hepatic growth factor (HGF), interleukin- (IL-) 10, IL-6, adenosine, kynurenic acid, TNF-*α*-stimulated gene 6 (TSG-6), heme oxygenase-1 (HO-1), IL-1 receptor antagonist (IL-1Ra), programmed death-1 ligand 1 (PD-L1), and galectins [33–36]. The mechanisms are illustrated (Figure 1).

### 2.1. Immunomodulatory Functions on Adaptive Immune Cells

The essential cell populations in the adaptive immune system are effector T cells, regulatory T cells (Tregs), and B cells. Abundant evidence supports a role for MSCs to exert immunoregulatory functions on T cells by suppressing proliferation and activation or regulating differentiation [15, 16, 22, 27, 35–48]. Murine BMSCs inhibit naive and memory T cell responses to their cognate antigens [37]. Murine BMSCs markedly suppress xenogeneic graft-versus-host disease (x-GVHD)-derived T helper (Th) 1 cells through adenosine accumulation [38] and also curb experimental autoimmune encephalomyelitis- (EAE-) derived Th17 cell activation in a CC chemokine ligand 2-dependent manner [27]. Recently, it is reported that human BMSCs-derived IL-1Ra can inhibit inflammation via suppressing the Th17 differentiation [39]. Interestingly, human BMSCs and murine BMSCs themselves can produce IL-17, but IL-17^+^ MSC displays an impaired immunosuppressive capacity [40]. Furthermore, BMSCs could induce Tregs and IL-10-producing T cells through the release of soluble factors such as PGE2, TGF-*β*, and HLA-G5 or cell-cell contact [41–43]. Moreover, human BMSCs can express Toll-like receptors (TLRs). Engagement of different TLRs in MSCs enhances their immunosuppressive properties by inducing IDO or impairing Notch signaling [46, 47]. Other MSCs, for example, UMSCs and AMSCs, can also significantly suppress T cell proliferation and activation [16, 44]. AMSCs inhibit the proliferative response and the production of inflammatory cytokines by antigen-specific CD4 and CD8^+^ T cells. Also, the numbers of IL-10-producing T cells and monocytes are significantly augmented upon AMSC treatment [16]. It is demonstrated that GMSCs suppress T cell proliferation and activation via interferon-*γ*- (IFN-*γ*-) induced stimulation of IDO and IL-10 [15]. Moreover, GMSCs ameliorate colonic inflammation by enhancing Tregs and IL-10 expression in mice [15]. GMSCs could significantly inhibit Th1 and Th17 cells and reduce the production of inflammatory cytokines (IFN-*γ*, IL-17A) via CD39/CD73 signaling [22, 45].

T follicular helper cells (Tfh) are recognized as crucial effector cells for B cell maturation and immunoglobulin production. UMSCs are found to suppress the differentiation of Tfh via the secretion of IDO [26, 49]. Most importantly, BMSCs can inhibit the Tfh response in lupus-prone mice [50]. Furthermore, it is suggested that BMSCs and UMSCs could indirectly affect the maturation and immunoglobulin production of B cells by inhibiting the Tfh immune reaction.

As we know, B cell development is dependent on the interaction of B cell progenitor and stromal cells, which provides a supportive microenvironment for B cells. BMSCs can suppress B cell proliferation, differentiation toward plasmablast, and immunoglobulin production dependent on galectin-9 or IL-1Ra signaling [51, 52]. BMSCs and AMSCs both suppress activation of blood B cells by phytohemagglutinin stimulation, but UMSCs do not display an inhibitory effect [53]. Besides, BMSCs could affect chemotactic properties of B cells, because chemokine receptors (CCR) and CXC chemokine receptors (CXCR), such as CXCR4, CXCR5, and CCR7, are decreased after B cell-BMSC coculture. However, B cell costimulatory molecules CD40, CD80, and CD86 and production of various cytokines are unaffected by BMSCs [54]. Interestingly, BMSCs do not inhibit B cell proliferation but only in the presence of inflammatory cytokine IFN-*γ* [55]. The suppressive effect of IFN-*γ* is related to its ability to stimulate the release of IDO by BMSCs, which in turn inhibits the proliferation of B cells [55]. Another group finds that enhanced autoantibody production is companied by increased plasma cells after BMSC administration [56].

Several years ago, a new regulatory subset called B regulatory cells (Bregs) was identified. These cells can interact with pathogenic T cells to inhibit harmful immune responses [57]. Transfer of UMSCs ameliorates experimental colitis by inducing Bregs [58]. AMSC treatment expands the Breg population in lupus mice *in vivo* [59]. Further study finds that murine BMSCs promote Bregs through stromal derived factor-1*α* (SDF-1*α*) and its receptor CXCR7-mediated signaling [60].

### 2.2. Immunomodulatory Functions on Innate Immune Cells

The innate immune system is the first line of host defense and consists of several types of immune cells including dendritic cells (DC), macrophages, natural killer T cells (NK), and mast cells (MC). MSCs exhibit potent immunomodulatory effects on these cells which may play an important role in the pathogenesis of pSS [5]. Human BMSCs can suppress activation and maturation of DC and impair their antigen-presenting ability through the release of TSG-6 [61–63]. Other research finds that the soluble factor IL-6 is involved in the immunomodulatory mechanism mediated by murine BMSCs through partial inhibition of DC differentiation [64]. Murine BMSCs impair TLR4-induced activation of DC resulting in downregulation of antigen presentation to T cells [65]. Human GMSCs can significantly blunt the maturation and activation of DC via PGE2-dependent mechanisms [66]. Human UMSCs can facilitate the shift of monocytes toward IL-10 producing phenotypes through the production of IL-6 and HGF [67].

MSCs reprogram macrophages into the anti-inflammatory M2 phenotype through the production of IL-6, granulocyte-macrophage colony stimulating factor (GM-CSF), PGE2, kynurenic acid, TSG-6, or IL-1Ra [52, 68–71]. Human BMSCs are activated by TNF-*α* to produce an anti-inflammatory mediator, TSG-6, and thereby create a negative feedback loop that attenuates inflammation by reducing TLR2-mediated signaling in resident macrophages [72]. MSCs have been shown to interact with NK cells dependent on their activation state. When resting NK cells are exposed to IL-2, the expression levels of activating receptors such as NKG2D, NKp30, and NKp44 are increased. Human BMSCs could inhibit IL-2-induced proliferation of resting NK cells, whereas they partially affect proliferation of activated NK cells. Human BMSCs also downregulated the activation and cytotoxicity of NK cells by mechanisms involving PGE2, IDO, TGF-*β*, HLA-G5, adenosine, or cell contact [43, 53, 73–75]. Interestingly, human NK cells secrete NAP-2 (CXCL7), a chemokine that can induce MSC migration to repair damaged tissue [76]. And GMSCs are capable of exerting suppressive effects on mast cells through the release of PGE2 and cell-cell contact both *in vitro* and *in vivo* [66, 77].

Recently, it has been thought that the immunomodulatory capabilities of MSCs are not constitutive but rather are subject to the inflammatory milieu or different signals. MSCs primed with IFN-*γ* or the stimulation of TLR signaling is required to induce their immunosuppressive phenotype [47, 55]. Proinflammatory cytokines such as TNF-*α* and IL-1 have been shown to enhance the effect of IFN-*γ* on MSC priming through the production of IDO or PGE2 [55, 78, 79]. The activation of TLR3 may promote the polarization of MSCs into immune-suppressive phenotype, while TLR4 stimulation may induce polarization of MSCs toward proinflammatory phenotype [80]. Therefore, MSCs show a plastic behavior and switch into an immunosuppressive phenotype depending on microenvironmental conditions.

## 3. BMSCs and Salivary MSCs Are Both Defective in pSS

Some evidence shows that BMSCs are defective in immunoregulatory functions in animal models and patients with pSS [81]. BMSCs have a significantly lower proliferative capacity and less osteogenic and adipogenic differentiation potentials in nonobese diabetic (NOD) mice. BMSCs derived from NOD mice fail to suppress T cell proliferation. CD4^+^Foxp3^+^Treg cells are much lower when splenocytes are cocultured with BMSCs from NOD mice. Furthermore, BMSCs obtained from pSS patients show an impaired suppressive effect on PBMC proliferative responses. Interestingly, activated T cells can induce BMSC apoptosis via the Fas/Fas ligand pathway [82]. IFN-*γ* synergistically enhances TNF-*α*–induced BMSC apoptosis [83, 84]. Furthermore, proinflammatory T cells inhibit the ability of BMSCs to repair damaged tissue. When the concentrations of IFN-*γ* and TNF-*α* are decreased, it significantly improves the function of BMSCs in repairing the tissue [83]. As we know, Th1 (IFN-*γ* and TNF-*α*)-mediated inflammation is a key feature in the salivary gland pathology [85]. T cells are activated in patients with pSS [86]. These findings suggest that T cell-induced BMSC apoptosis may contribute to BMSC impairment in pSS. Collectively, these results demonstrate that the biological and regulatory functions of BMSCs are impaired in pSS [81].

Recently, MSCs have been also found in the salivary glands of patients with pSS. Such organ-specific MSCs may have advantages for treatment of the specific tissue of origin since they could directly act on the target cell. Jeong et al. [87] efficiently isolates and amplifies MSCs in large amounts from human parotid and submandibular salivary glands *in vitro*. These cells express the same characteristic MSC markers; they are negative for hematopoietic stem cell and salivary gland epithelium markers. They are able to differentiate into adipogenic, osteogenic, and chondrogenic cells and notably into amylase-expressing cells. Transplantation of SGSCs restores salivary gland function in radiation-damaged rat salivary glands [87]. However, MSCs from the labial glands of patients with pSS have a deficiency in salivary gland-like cell differentiation [88]. Taken together, BMSCs and salivary MSCs are both defective in patients with pSS disease condition, meaning that it is not ideal to treat patients using their own MSCs. We may use allogeneic MSCs for the treatment of pSS, due to nonimmunogenic characteristics and low or absent expression of non-major histocompatibility complex- (MHC-) I and MHC-II [12].

## 4. The Application of MSCs in the Treatment of pSS

pSS is a disease triggered by the breakdown of self–nonself discrimination and a subsequent autoreactive immune response. Salivary gland pathology is mainly a Th1-mediated immune reaction [85]. Th17 and Tfh cells are also associated with inflammation and clinical profiles in pSS [49, 86]. The Treg percentage is altered in the peripheral blood [89–91], and their suppressive function is compromised in pSS [92]. In contrast, another group reports that the Treg subset did not change in patients with pSS [93]. Therefore, the imbalance of these immune cells contributes to the pathogenesis of pSS. It is clear that MSCs could possess potent immunomodulatory functions on both adaptive and innate immune cells and especially reset the immune imbalance by upregulating Tregs and downregulating Th1, Th17, or Tfh cells. In addition, MSCs can differentiate into salivary epithelial cells, presenting an option as a suitable alternative treatment for pSS. The unique immunomodulatory and biological properties of MSCs make them candidates for cell therapy to repair tissue and organ damages caused by pSS. We have summarized the applications of MSCs in the treatment of patients with pSS or in animal models (Table 1).

MSC treatment ameliorates sialadenitis in the mouse model and in patients with pSS partly through reducing the proliferation of T cells, decreasing Th1, Th17, and Tfh cells, and increasing Tregs [44, 49, 81, 94–98]. The pSS patients tolerate the allogeneic UMSCs well, have an improvement in symptoms and a decrease in serum anti-SSA/anti-SSB antibody without significant adverse events [81]. Interestingly, infused allogeneic BMSCs could migrate toward the inflammatory regions in an SDF-1-dependent manner, as neutralization of SDF-1 ligand CXCR4 abolishes the effectiveness of BMSC treatment. Therefore, allogeneic BMSCs might target local sialadenitis and systemic inflammatory responses in pSS patients by a mechanism that is dependent on SDF-1/CXCR4 signaling [81]. Another team finds that treatment with BMSCs prevents a decline in the salivary flow rate and lymphocytic infiltrations in the salivary glands of NOD mice [94]. Furthermore, BMSCs enhance tear production in the NOD mouse model, due to decreased inflammation and increased expression of aquaporin 5 [95]. Although the number of lymphocytic foci in the lacrimal glands of treated animals did not change, the size of the foci decreased by 40.5% [95].

As we know, bone marrow mesenchymal cells can be easily contaminated with hematopoietic cells. Researchers isolate CD45^−^/TER119^−^ cells from murine bone marrow by depleting CD45^+^ cells or TER119^+^ hematopoietic cells. CD45^−^/TER119^−^cells are identified as BMSCs because they are positive for stem cell surface markers and have multiple differentiation potentials. Treatment with CD45^−^/TER119^−^cells could prevent loss of saliva flow and reduce lymphocytic infiltrations in SG of NOD mice through downregulation of inflammatory cytokines such as TNF-*α*. Notably, the infiltrations of T and B cells are decreased in all foci, while the frequency of Tregs is increased. Investigators speculate enhanced Tregs inhibit the inflammation in SG. Meanwhile, fibroblast growth factor-2 (FGF-2) and epidermal growth factor (EGF) involved in the growth, regeneration, and maintenance of salivary glands are both increased after CD45^−^/TER119^−^ MSC treatment. This suggests the CD45^−^/TER119^−^ BMSCs are effective in both preventing deterioration of saliva secretion and reducing lymphocytic influx in salivary glands with a systemic effect [96].

Tfh cells are recognized as crucial for B cell maturation and differentiation. Unlike BMSCs, UMSCs are found to suppress the differentiation of Tfh cells via the secretion of IDO in patients with pSS [49]. Therefore, UMSCs could provide a novel therapeutic approach for pSS by targeting Tfh cells. Other investigators find that human UMSCs inhibit proliferation of healthy T cells, but not T cells from pSS patients. Interestingly, they develop a new microencapsulation technique to make a coculture system separating UMSCs from T cells. This approach avoids a systemic immune reaction in the host. The specialized UMSCs could suppress pSS T cell proliferation and restore the Tregs/Th17 ratio, suggesting a drug delivery system able to enhance the immunomodulatory effects of UMSCs in pSS [44].

A small preclinical study regarding the application of AMSCs shows that when allogeneic AMSCs are implanted around the lacrimal glands in 12 dogs (24 eyes) with refractory keratoconjunctivitis sicca (KCS), improvement is observed in the Schirmer tear and ocular surface integrity tests during nine months follow-up. No systemic or local complications appear. However, a caveat is that no control cells are mentioned in this study [97].

Interestingly, it has been demonstrated that human BMSCs could temporarily change into salivary gland epithelial cells (SGEC) in a coculture system. BMSCs have comparable cellular structures and expressed several salivary genes such as aquaporin 5, E-cadherin, and *α*-amylase (*α*-AMY) when cocultured with SGEC. These BMSCs can secrete *α*-AMY. Moreover, they have comparable cellular structures to SGEC, such as tight junctions and numerous secretory granules, as shown by electron microscopy [99]. Some proteins such as ankryin-repeat-domain-containing-protein 56, high-mobility-group-protein 20B, and transcription factor E2a are the putative regulatory factors involved in the transdifferentiation of BMSCs into SGEC in an animal study [31]. These data suggest that cocultured BMSCs can generate a salivary gland acinar phenotype, meaning that this approach has potential application to treat salivary gland diseases such as pSS [31, 32, 99]. However, we still do not know whether pSS disease-derived BMSCs switch into acinar epithelial cells. Further study is needed to answer this question.

## 5. Perspectives: Learn from Bioengineering Strategies

In order to improve the function of MSCs, novel methods such as retroviral transduction, electroporation, or CRISPR-associated protein-9 nuclease (Cas9) of foreign genes have been utilized to engineer MSCs [100–103]. In cancer, MSCs have been successfully modified with a tumor suppressive gene to inhibit progress or metastasis of tumor cells. Similarly, current bioengineering strategies could be applied in pSS. For instance, IL-10 is a powerful cytokine to suppress inflammation. One team engineered mouse BMSCs with an IL-10 gene (called IL-10-BMSCs) through retroviral transduction. They demonstrated that IL-10-BMSCs, but not BMSCs alone or PBS, could modulate inflammatory arthritis and decrease the histological scores [100]. Besides, MSCs transfected with minicircles encoding CXCR4 are more likely to migrate to the injury site [102]. Moreover, it is possible to track the MSCs which are transfected with minicircles and monitor their elastic properties with noninvasive microscopy technologies. Recently, a novel gene-editing technology based on a bacterial CRISPR-associated protein-9 nuclease (Cas9) has been successfully used to target important genes in many cell lines and organisms [103]. It may be possible to develop a successful MSCs-derived therapy for pSS if we target crucial genes involved in MSC immunomodulation, such as soluble factor production and chemokine receptors using Cas9 methods.

Recently, extracellular vesicles (EVs) have been considered as a functional molecule with their potential for treatment of pSS. As we know, extracellular vesicles are secreted by many cells, including MSCs, and are classified into two types of particles: exosomes, with a size of 50 to 100 nm derived from the endosomal compartment, and microvesicles (MVs), with a size between 100 nm and 1000 nm. It has been reported that BMSCs, UMSCs, and AMSCs may be a suitable source for therapeutic EVs [104–107]. EVs can mediate cell-to-cell communication and participate in many processes including inflammation, proliferation, cell differentiation, and immune signaling [106, 108]. They can also act directly with the target cell membrane by fusion, transferring components into intracellular compartments or causing endocytosis. MVs have a role in antigen presentation and activation of endosomal receptors such as Toll-like receptors [109]. EVs can carry autoantigens, cytokines, and tissue-degrading enzymes. Collectively, EVs could repair damaged tissue or serve as agents for drug delivery in autoimmune disease [106, 108, 110]. Recently, it has been reported that MSCs derived from human-induced pluripotent stem cells (MSCs-iPSCs) can prevent the onset of sialadenitis in NOD mice. Furthermore, EVs derived from MSCs-iPSCs are shown to suppress activation of immune cells *in vitro*. And the infusion of these EVs at the predisease stage reduces the lymphocyte infiltration in salivary glands and serum autoantibody levels in NOD mice [111].

We focus on the therapeutic effect of GMSCs in autoimmune disease, especially in pSS. GMSCs are relatively easy to isolate, homogenous and proliferate rapidly *in vitro*, and have a strong immunomodulation via suppression of Th1 and Th17 cells and enhancement of Treg differentiation [22]. We further find that GMSCs suppress human T cell-mediated diseases in the x-GVHD model via CD39/CD73/adenosine and IDO signals [112]. In addition, GMSC populations existing within the inflamed gingival tissue are functionally equivalent to those derived from healthy gingival tissue, indicating the possibility of treatment with the patient's GMSCs [113]. Furthermore, our unpublished data have documented that GMSCs could inhibit the proliferation and IFN-*γ* production of peripheral T cells from patients with pSS *in vitro*. Next, we plan to study whether the patient with pSS will benefit from GMSC transfusion.

There remain many challenges to overcome for clinical application of MSCs in pSS. First, there are great variations in the MSC isolation protocols, culture systems, MSC dose, cell delivery methods, and transfusion frequency in the reported studies. Secondly, the quality control of diverse MSCs is not defined. It will be necessary to establish standardized protocols for cell culture, differentiation, and expansion, as well as recommended transfusion schedules and evaluation of responses. Thirdly, the number of enrolled patients reported in published papers is small and may not be sufficient to provide a reliable conclusion. Fourthly, another restriction in the field of clinical application of MSCs is the biosafety issues relevant to tumorigenicity [114]. Some papers report that MSCs enhance tumor angiogenesis and promote tumor formation in mice [115, 116]. Finally, whether MSC transfusion will have a long-term effect in pSS is unknown.

## 6. Conclusions

In summary, MSCs can be obtained from different sources and are especially abundant in adipose and dental tissues. They possess potent immunomodulatory functions and act on both adaptive and innate immune responses. They may repair damaged tissue via suppressing Th1/Th17/Tfh cell responses and upregulating Tregs. Furthermore, MSCs could induce Breg cells and modulate innate immune cells such as DC, macrophages, mast cells, and NK cells. Moreover, new bioengineering approaches, such as Cas9 methods, may improve the function of MSCs. Currently, there is no curative clinical therapy for pSS, so MSCs-based therapies show great potential in this area, with the expected capacity to significantly suppress the inflammation and preserve salivary function in pSS. As of now, no conclusive evidence to support the use of MSC-based therapies has been published. In the near future, randomized controlled trials of the therapeutic use of MSCs in pSS will be of considerable interest.

## Figures and Tables

**Table 1:** Application of MSCs in treating animal models and patients with pSS.

Types of MSCs	Treatment		Recipient (human)	Recipient (mice)	Follow-up	Effect	Outcome	Reference
	Cell numbers, origin	Administration						
BMSCs	1 × 10^5^, one dose, BALB/c or B6 mice	IV injection at 6 (prevention group) or 16 weeks age (treatment group)	NA	NOD mice	8 weeks or 18 weeks	Inflammatory area ↓ in SG, salivary flow rate ↑, Tregs ↑, Th2 ↑, Th17 ↓, Tfh ↓	Improvement	[81]
UMSCs	1 × 10^6^/kg, one dose, human	IV injection	Patients with pSS	NA	12 months	SSDAI and global assessment Vas score ↓, unstimulated and stimulated salivary flow rate ↑, anti-SSA/Ro and anti-SSB/La ↓	Improvement	
BMSCs	5 × 10^5^, four doses, mouse	IV injection	NA	NOD mice	4 weeks	Saliva flow rate ↑, lymphocytic infiltrations ↓, serum IFN-*γ* ↓, IL-10 ↑, TGF-*β* ↑	Improvement	[94]
BMSCs	1 × 10^6^, one dose, mouse	IP injection	NA	NOD mice	4 weeks	Tear production ↑, aquaporin 5 mRNA ↑, lymphocytic score did not change	Improvement	[95]
CD45^−^TER119^−^ BMSCs	1 × 10^7^, four doses, human	IV injection	NA	NOD mice	14 weeks	Salivary flow rate ↑, lymphocytic infiltration in SG ↓, T and B cells ↓, Tregs ↑ in all foci	Improvement	[96]
UMSCs	Human	Coculture	pSS	NA	NA	Differentiation and proliferation of Tfh ↓	Suppression	[49]
UMSCs microencapsulated	Human	Coculture	PBMC from 10 pSS	NA	NA	Proliferation of T cell ↓, Th1 ↓, Th17 ↓, Tregs ↑	Suppression	[44]
AMSCs	8 × 10^6^, allogeneic, one dose, per eye, dog	Locally injected around lacrimal glands and gland of eyelid	NA	Dog with KCS	9 months	Schirmer tear test ↑, ocular parameter score ↓	Improvement	[97]
